# Intermediate ice scour disturbance is key to maintaining a peak in biodiversity within the shallows of the Western Antarctic Peninsula

**DOI:** 10.1038/s41598-021-96269-9

**Published:** 2021-08-18

**Authors:** B. J. O. Robinson, D. K. A. Barnes, L. J. Grange, S. A. Morley

**Affiliations:** 1 National Oceanogrpahy Centre Southampton, University of Southampton, European Way, Southampton, SO14 3ZH UK; 2 British Antarctic Survey, National Environment Research Council, High Cross, Madingley Road, Cambridge, CB3 0ET UK; 3 School of Ocean Sciences, Bangor University, Bangor, Gwynedd LL57 2DG UK

**Keywords:** Ecology, Structural biology, Climate sciences, Ecology, Environmental sciences, Ocean sciences

## Abstract

Climate-related disturbance regimes are changing rapidly with profound consequences for ecosystems. Disturbance is often perceived as detrimental to biodiversity; however, the literature is divided on how they influence each other. Disturbance events in nature are diverse, occurring across numerous interacting trophic levels and multiple spatial and temporal scales, leading to divergence between empirical and theoretical studies. The shallow Antarctic seafloor has one of the largest disturbance gradients on earth, due to iceberg scouring. Scour rates are changing rapidly along the Western Antarctic Peninsula because of climate change and with further changes predicted, the Antarctic benthos will likely undergo dramatic shifts in diversity. We investigated benthic macro and megafaunal richness across 10–100 m depth range, much of which, 40–100 m, has rarely been sampled. Macro and megafauna species richness peaked at 50–60 m depth, a depth dominated by a diverse range of sessile suspension feeders, with an intermediate level of iceberg disturbance. Our results show that a broad range of disturbance values are required to detect the predicted peak in biodiversity that is consistent with the Intermediate Disturbance Hypothesis, suggesting ice scour is key to maintaining high biodiversity in Antarctica’s shallows.

## Introduction

Disturbance events occur in almost all natural ecosystems and tend to be a significant driver, influencing assemblage diversity, structure and function^1^. However, the disturbance literature is divided. Some studies conceptualise disturbance as departures from a ‘stable’ state^2,3^ and detrimental to biodiversity^4^, whereas, others present cases where disturbance maintains high biodiversity^5^ and promotes resilience to further change^6^. This paradox can be addressed with the Intermediate Disturbance Hypothesis (IDH), which posits stable coexisting states under “intermediate” disturbance conditions where species diversity is predicted to be highest^7,8^*.* The IDH itself however is disputed on both theoretical and empirical grounds, with studies rarely finding the predicted peaked relationship^9,10^. Literature that has found evidence for peaks in diversity include successional, post-iceberg disturbance studies^11,12^ and theorectical models^13^. In contrast, a meta-analysis of disturbance studies found that a key factor in the detection of species richness peak was the inclusion of a broad range of disturbance levels, which has not been achieved in the majority of empirical studies^14,15^. The Western Antarctic Peninsula has one of the largest disturbance gradients on earth^16^ and is considered a hotspot of benthic diversity^17–19^, making it an ideal natural laboratory for analysing the relationship between disturbance and diversity.

The shallow Antarctic seafloor (< 40 m depth) is home to one of the most naturally disturbed assemblages, due to frequent iceberg scouring disturbance^16,20^. Ice scour disturbance, defined as when the keel of an iceberg impacts the seafloor, are distinct events in both time and space^21^ resulting in high mortality of > 98.5% for macro and megafauna^22,23^.Here, we consider any contact of ice with the seafloor that results in scour as ice scour disturbance, the majority of disturbance recorded here are likely caused to be small to large ice bergs^24^. The frequency of ice scour disturbances varies due to bathymetry, latitude and topography with the highest frequency in the shallows; at some sites > 35% seabed is scoured per year at 5 m depth^25^ . Typically ice scours are limited to ~ 500 m depth, though they may rarely occur deeper^26,27^. Ice scour is the key factor driving biodiversity and structure in the Antarctic shallows^21,28–31^ . However, its influence has been little explored between 40 and 100 m despite this depth range being an area of significant change in ice scour frequency^32^, so a broader study between 10 to 100 m depth is required.

In recent decades, there have been drastic shifts in the cryosphere through atmospheric and marine warming due to greenhouse gas-driven climate change^33–36^. This is particularly true along the Western Antarctic Peninsula (WAP)^37^, a hotspot of regional physical change^38^. In the Western Antarctic the seasonal sea-ice maximum area and duration have reduced over the last four decades^39^ (although the signal is noisy). As a result, there has been an increase in iceberg movement (because of less time locked into seasonally frozen sea ice), increasing the frequency of ice scour impacts (~ 0.6 scours for each day of sea ice loss at 10 m depth)^32^. Increasing numbers of glaciers and ice shelves in retreat (87% along the WAP)^33^, have led to high rates of iceberg calving^32^, where rates of ice scour across all depth ranges are likely to increase substantially over the next century^32,40^. Longer-term predictions estimate there will be an eventual decrease in ice scour events as glaciers pass the grounding line and retreat onto land^40–43^.

Understanding how marine ice losses and ice scour will change the ecology of the Antarctic benthic macrofauna is key to understanding the future of this ecosystem^1^, and provides insights into disturbance ecology. Disturbance is a heavily debated topic, and despite progress in this field, there is a lack of consensus on how this impacts systems when disturbance ranges move outside the historical norms^44^. It is proposed through the Intermediate Disturbance Hypothesis that within a broad range of disturbance, species richness is maximised at intermediate levels due to competitively inferior, disturbance-tolerant species and competitively dominant, disturbance-sensitive species coexisting^7,8^. However, many reports, which have been critical of the Intermediate Disturbance Hypothesis, only test the diversity-disturbance relationship across a small range of potential disturbance values^14,15^ or struggle to isolate relative, legacy and absolute disturbance^2,25^. Therefore, sampling macro and megafaunal assemblages across one of the largest disturbance gradients on Earth, occurring over a small spatial scale, provides an ideal opportunity to test Intermediate Disturbance Hypothesis, and investigate relationships between disturbance and biodiversity. Furthermore, the fauna itself is data poor, between 40 and 100 m depth, probably due to poor overlap of sampling methods at this depth range^32^. Gathering comprehensive data from this assemblage before further climate-driven disturbance change is essential, if we are to understand the impacts of long-term change in this environment.

We surveyed benthic macro and megafaunal samples across 100 m depth from three sites on a steeply sloping marine rocky shore on Adelaide Island, WAP (67°35′ S, 068°07′ W**,** Supplementary materials, Figs S1). Most Antarctic species are relatively long-lived with extremely slow growth, reproduction and movement when compared to lower latitudes^45,46^. It follows that these taxa are particularly good indicators of ice scour disturbance, with some recovery times predicted to be decades long (although exception exists^47^). The broad ranges of disturbance regimes provide an opportunity to test disturbance-biodiversity relationships, within a similar environment and provide insights into the likely fate of the Antarctic benthos as they undergo dramatic disturbance changes over the next century. In this study, we aim to describe the patterns in macro and megafauna biodiversity from 10 to 100 m depth using multivariate anaylsis and then compare mulitple diversity indicies against the disturbance gradient, alongside mulitple other environmental variables using mulitple regression modelling. If ice scour is a driving influence behind biodiversity within the shallow Antarctic benthos, linear and polynomial regressions will be used to assess with the disturbance-biodiversity relationships are congruent with the IDH.

## Methods

### Study area

The study area was steeply sloping rocky shores (67°35′ S, 068°07′ W) around Ryder Bay, Adelaide Island, Western Antarctic Peninsula between 10 and 100 m depths. Three sites were selected along the North coast of Ryder Bay, with similar topography (Supplementary Materials S2) and exposure to predominant current flow and iceberg scour, providing homogenous conditions. Adjacent to these sites, the Rothera Time Series (RaTS)^48^ provided long-term (since 1997) oceanographic measurements across all sample depths including light levels, temperature, salinity and standing stocks of phytoplankton.

### Ice Scour

Ice scour is directly measured in the shallows around Rothera and Carlini stations in Antarctica, but the density of deeper scours is surveyed using ship-borne multibeam echo sounding. Where measured, ice scour occurrences are high^1,6,7,9^ and there has been a dramatically increased shift in density and/or frequency within the top 100 m^21,27,29^. Our ice scour counts were collected through analysis of scours per square kilometre in multibeam bathymetry from the JR17001 (ICEBERGS1) cruise around Ryder Bay^44^, between 0 and 500 m depth. Raw counts showed large variations in absolute values. Therefore, a log transformation was used to constrain the data range. An asymptotic regression curve (supplementary material, S3) provided the best fit for the data. Ice scour disturbance values between 10 and 100 m were then interpolated from this regression model.

### Environmental factors

Environmental variables were collated from the Rothera time series (RaTS). As Antarctic macro and megafauna can be very long lived^45,46^, this RaTS long-term data were used to describe the ambient environment experienced by the study taxa. All RaTS data were averaged across month to ensure even representation of the annual variation from 2011 to 2018. Maximum temperature range was calculated as the maximum and minimum recorded temperature from all 7 years at each specific depth. Benthic growth was calculated from bryozoa and serpulidae (spirobid worm) growth ring analysis^49^ from 5 to 500 m depth. Bryozoa growth is considered to represent a median value for growth across all benthic taxa^50^. A quadratic spline curve provided the best fit for the data; from this, we interpolated values for each 10 m depth interval across our study area (Supplementary material on spline regression, S4).

### Macrofauna

Samples were collected at every 10 m depth interval between and including 10–100 m depth from 3 sites along Ryder Bay for a total of 30 stations. At each site the macrofauna assemblage and substrate were surveyed between February 2016 and June 2016, through 50 replicate images per station recorded via ROV, giving 1500 samples in total. A modified DeepTrekker DTG2 was used to collect images and sample morphotypes (more details in supplementary material S5). Species accumulation curves were constructed for each station to ensure representation of rare species.

For each sample, a random area of seabed was selected and photographed (approximately 1.5 m^2^). Images were corrected for lens distortion with *Hugins* photo editing software and cropped to remove areas with insufficient detail or those that were beyond the focal plane of the image. Macrofauna within the image were counted and identified into morphotypes. Specimens collected were later used to aid species identification and increase taxonomic resolution (188 specimens collected). Sample area could not be quantified as the seafloor was not uniform in shape, structure or composition. Attempts were made to ensure sampling was as uniform as possible and all images were scaled using two lasers but there remains an unquantifiable variability across each sample.

### Data analysis

Biodiversity was expressed as species richness, the number of macrofaunal species present within a sample, Shannon-Weiner index^51^ and Fisher’s α^52^. Shannon-Weiner and Fisher’s α were analysed as Shannon-Weiner includes an evenness measure and Fisher’s α is independent of sample size, to ensure that neither evenness nor sample size significantly alter the results. We preformed linear and polynomial (quadratic and cubic) regression analyses to determine the best-fit shape of biodiversity-disturbance relationship. Variance Inflation Factors (VIF) were used to identify any collinearity (VIF values between 1 and 5 = moderately correlated and > 5 = highly correlated^53^). Parameters of regression were estimated using R package *lme4* with Loess smoothing using the R package *ggplot2* to assess potential nonlinearity between biodiversity and disturbance. All statistical analyses were preformed using R 3.5.2 and Minitab 19.

Macrofauna composition was analysed using Primer 7 (version 7.0.17). Taxa abundance was transformed using square root function to reduce the influence of hyper-abundance and non-metric multidimensional scaling (nMDS), using a Bray–Curtis resemblance matrix was used to compare macrofaunal composition across all depths and sites. SIMPER (SIMilarity PERcentages) analysis was used to calculate the contribution of each taxa to group similarity, across the different factor levels.

## Results

Depths between 10 and 30 m were dominated by mobile grazers such as by *Nacella concinna* (limpets) and *Sterechinus neumayeri* (sea urchins). These depths were also coincident with the highest prevalence of algae, although coralline algae was still found in reasonably high frequencies at 60 m depth. Between 40 and 50 m depth, a mixed assemblage of sessile suspension feeders and mobile grazers/scavengers were dominant with species such as *Cnemidocapra verrucosa* (solitary ascidian), *S. neumayeri* and *Ophionotus victoriae* (brittle star). At 60–100 m depth, sessile suspension feeders dominated with some associated fauna, groups of Porifera and Bryozoa in particular. Due to bryozoans only being identifiable to species level under a microscope, multiple collections were made and found two bryozoan morphotypes represent multiple species. Bryozoan diversity is likely under reported but did coincide with the species richness peak between 50 and 60 m depth. Suspension feeders included, *Neofungella* sp. (Stenoleamate byrozoan), *Perkinsiana littoralis* (feather worms) and *Anoxyclalyx joubeni* (structure-forming hexactinellid sponge).

No clear zonation was observed between 10 and 100 m depth; rather a gradual shift between assemblages with a broad overlap in species ranges (Supplementary materials Figs S6). Across all depths the assemblage composition showed large degree of variability or ‘patchiness’, typical of Antarctic benthos and the resulting from spatial heterogeneity in iceberg scours^54^. Gastropoda, Asteroidea and Anthozoa groups showed no depth trend with individual taxa having wide depth ranges, although Actiniaria (sea anemones) tended to be found deeper (> 60 m depth, but heavily species specific). Bryozoa, Ascidia and Porifera were found deeper, with the exception of *Beania* sp. (Ctenostomata Bryozoa) and *Cnemidocarpa verrucosa*. *Sterechinus neumayeri* and *O. victoriae* had a notable prevalence across all depths, although these taxa were found in higher abundance at depths shallower and deeper than 50 m, respectively. Representatives of the Holothuroidea (sea cucumbers), Hydrozoa and Entoprocta were more prevalent at intermediate depths (30–70 m).

**Figure 1:**
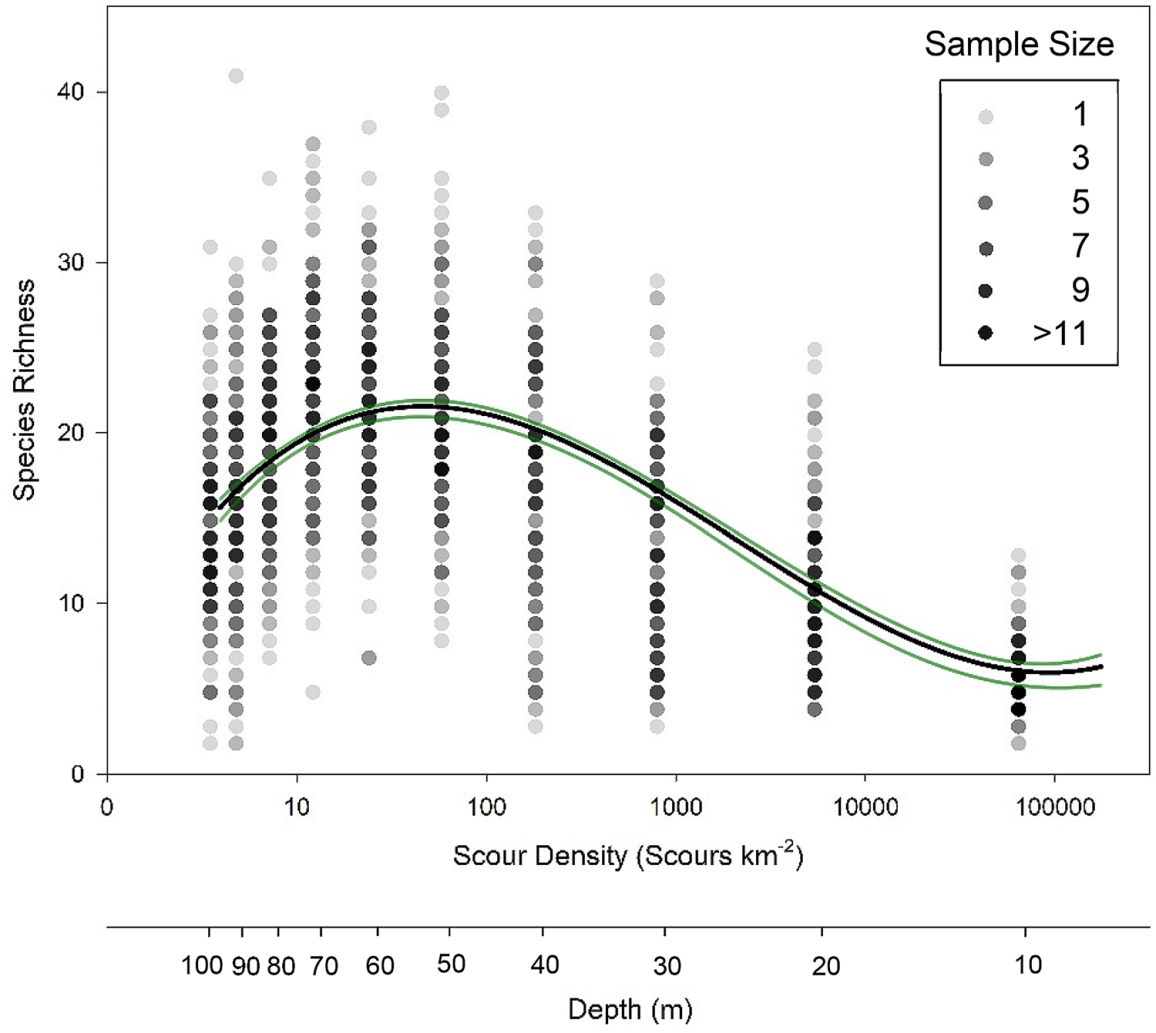
Relationship between species richness and disturbance. Line (model) of best fit was non-linear regression (cubic model, black line). Points are samples, with increasing shades of grey representing a greater number of samples. Green lines are 95% confidence intervals. Total sample number is 1500, evenly divided across 10 m depth intervals. Plot constructed in RStudio v1.1.463, https://www.rstudio.com/.

Counts of scours per square kilometre on seabed mapping (vessel multibeam) data spanning Marguerite Bay showed that ice scour disturbance varied considerably across all depths. Scour density decreased from 1.75 × 10^5^ scours per square kilometre at 10 m depth to 3.92 scours per square kilometre at 100 m depth. Species richness showed a peaked relationship with study depth. We found an average species richness of 5.77 per image at 10 m depth, increasing to 22.49 between 50 and 60 m depth, before decreasing to 14.77 species richness by 100 m depth. The peak in species richness coincided with 32 scours per square kilometre. Linear and polynomial regression analysis found a cubic function (*F*_*3,1496*_ = *385.94, r*^*2*^ = *0.44, p* < *0.01*) and provided the best-fit relationship between biodiversity and disturbance (Fig. 1). The regression line shows a clear unimodal relationship, with a wide range of species richness at each level of disturbance. The maximum range of species richness at each depth was on average, 28.2 species (*average Standard Deviation 5.43,* across all depths). We found similar diversity-disturbance trends with all diversity indices (Supplementary materials Figs S7). As Shannon-Weiner (r^2^ = 32.6, p < 0.001) and Fisher’s α (r^2^ = 26.7, p < 0.001) diversity indicies had lower r^2^ values than the relationship between depth and species richness (r^2^ = 48.7, p < 0.001), further analyses used species richness.

Growth data for selected macrofauna interpolated from the literature^49^ rose from 0.08 g day^−1^ m^−2^ at 10 m to peak at 0.14 C g day^−1^ m^−2^ at 40 m depth, decreasing to 0.12 g day^−1^ m^−2^ at 100 m depth (supplementary material S8). Growth correlates with ice scour disturbance (*VIF* = *1.68*) and therefore has a quadratic correlation with species richness (*F*_*2,1497*_ = *291.20, r*^*2*^ = *0.279, p* < *0.001*). However, the maximum range of values for growth between 40 and 100 m depth, was 0.02 g day^−1^ m^−2^. These values are below the signal noise threshold, of 0.05 g day^−1^ m^−2^, and cannot be distinguish from experimental error. Average annual salinity varied by a maximum 0.54‰ across all stations, which is in line with previous work on coastal Southern Ocean salinity being stable and constant throughout the year (except in the intertidal zone)^55^ (supplementary material S5). The range of growth and salinity were not considered large enough to detect any correlation with species richness, so were removed from the analysis.

Average annual sea temperature was − 1.04 °C at 10 m depth. This variable decreased to a minimum of −1.09 °C at 25 m depth, before increasing to −0.73 °C at 100 m (supplementary material S3). Average annual sea temperature was correlated with ice scour disturbance (*VIF* = *1.78*) but did not correlate with species richness. Maximum sea temperature range at 10 m depth was 4.00 °C, which decreased exponentially with depth, reaching 2.71 °C at 100 m depth (supplementary material S3). Chlorophyll α concentration decreased at an exponential rate with depth from 1.85 mg m^−3^ at 10 m to 0.16 mg m^−3^ at 100 m, as did photosynthetically active radiation, from 47.70 to 0.18 µmol m^−2^ s^−1^ (supplementary material S3). Sea temperature range, chlorophyll α and light levels exponentially decreased with depth, with the majority of change occurring in the top 20 m depth. All variables had a strong collinearity with scour density (*VIF* = *20.09, 205.47, 25.24 respectively*) and were therefore removed from the model. Linear and polynomial regression analyses for sea temperature range, chlorophyll α and light levels had a similar unimodal relationship, as ice scour disturbance. However, all environmental variables had lower r^2^ values and poorer overall fit, particularly past 30–40 m depth. In addition, there were only small differences between sites, and the inclusion of sediment and site did not significantly improve the model (Supplementary information on multiple regression analysis S4). These were tested to account for variation in ice abundance, topography and current between all three sites.

## Discussion

The Antarctic marine shallows are home to one of the largest natural disturbance gradients on earth, up to 100% mortality across the entire macrobenthic population within the intertidal (with some exceptions see Waller, et al. ^56^), to near 0% mortality^21^ around 200 m depth^57^. Shallower than 40 m depth ice scour disturbance is a key controlling factor^21,28–30^ as only disturbance resilient species are able to persist, reducing species richness^7,8^. However between 40 and 100 m depth there is little information on which environmental factors influence the Antarctic benthos and furthermore what species occupy this depth range^32^. Deeper than 40 m we found a unimodal relationship between macro and megafauna species richness and ice scour disturbance, with a peak in species richness at intermediate levels of ice scour disturbance. This concurs with the Intermediate Disturbance Hypothesis, a widely recognised concept, but one that has produced many reviews and critiques^15^.

The disturbance-diversity pattern identified across our depth range showed an extreme variability in species richness across all depths. This patchiness is suggestive of ice scour disturbance being the driving factor, as a spatially and temporally discrete mass mortality event^22,23^. The variation in species richness amongst samples from similar depth likely reflect a patchwork of assemblages at different stages of recovery, from previous ice scour events. However, ‘patchiness’ (or spatial heterogeneity) was lowest at 10 m depth, which was dominated by a mobile assemblage, which could rapidly re-invade recent iceberg scours, the impact of ice scour impacts across a wider area, enough to homogenise the fauna at this depth^25^.

The influence of other environmental variables could not be completely isolated from disturbance, although many of them showed minor changes beyond 30 m depth. Additionally, we do not know at what depths lower thresholds of disturbance are reached and species richness starts to be controlled by other factors. Likely the flux of food particles from the surface, which much of the Antarctic seafloor community is reliant on^58^, will become a crucial factor at depth. For example, Jansen, et al. ^59^ showed that the abundance and richness of types of benthic fauna could be predicted by food availability at depths below 200 m. We could not confirm any influence of light level or chlorophyll a concentration on biodiversity; however, they are likely to play a major, but perhaps complex, role in the structuring of benthic biota and ecosystem dynamics^60^, particularly below the depth of peak biodiversity.

The Western Antarctic Peninsula is a climate change hotspot that is predicted to warm if current emissions continue^61^. This change is also likely to result in a profound impact on ice scour disturbance, as glaciers continue to retreat and sea-ice reduces in both extent and duration^33–35,41^. Over the next century icebergs are likely to calve at an increased rate and with higher mobility as they are less likely to be held in place by seasonal sea ice^40^. As argued in this study, ice scour disturbance is a key controlling factor down to 100 m depth; if disturbance regimes continue to change, we expect benthic biodiversity to alter considerably.

We suggest two potential futures within the next century for biodiversity in the shallows, based on the diversity-disturbance patterns reported in this study and the current composition of the Antarctic macro and mega-fauna. First, if scour disturbance increases rapidly the macro and megafaunal assemblage will struggle to redistribute, particularly if these species ranges are restricted by depth-dependent environmental and biological factors. The majority of macro and megafaunal species are long lived with slow growth, locomotion and reproduction, when compared to lower latitudes^45,46^ (but may grow faster with moderate warming^62^). Within this context, a century may not be long enough for these species to migrate away from, or adapt to, new conditions. Increasing ice scour is expected to remove many of the competitively-dominant, disturbance-sensitive species, such as *Mycale acerata* (sponge), which have slow growth and reproduction rates^63^. However, many macro and megafauna species have wide depth-ranges (*M. acerta* for example between 20 and 90 m depth) and so although species richness is controlled by ice scour in the shallows, species may still exist at extremely low frequencies across a wide spectrum of disturbance levels.

The presence of species across a wide depth gradient, may allow a few individuals found at the extremes of their ranges to thrive as conditions shift in their favour. The broad depth-ranges of many species support a second prediction, that the increase in ice scour disturbance would redistribute species into deeper waters, as the diversity migrates in response to a new disturbance pattern. The second prediction is based on biodiversity being driven by disturbance, even at the deeper end of our depth range. Beyond 100 m depth the relative difference in disturbance is minute and it is likely that primary production (more specifically bloom duration^41^) will be the limiting factor, restricting the depth over which these species can redistribute. However, the pattern between sea-surface chlorophyll and species richness is multifaceted, with trophic dependent relationships and dependent on multiple physical variables^59^. This study cannot disentangle where, or if, the relative contribution of disturbance is surpassed by primary production as a driving factor and instead asserts that between 60 and 100 m depth the influence of disturbance is likely to wane.

With both predictions, we can expect species richness loss in the shallows (10–30 m) as disturbance tolerant species reach their limit and either redistribute to deeper waters or are extirpated. Both of the predictions made here are by no means mutually exclusive, there may well be a drop in diversity across all depth ranges, as species are unable to move outside of their established ranges, combined with a shift in the now reduced biodiversity peak, as the intermediate levels of disturbance shift deeper. The eventual limit of the depth shift in biodiversity will likely be dictated by the depth related reduction in primary production^64^. However, climate change-induced sea ice changes and associated changes in light regime^65^ are predicted to increase bloom duration^41^ potentially allowing more species to persist at a greater depth.

In particular, species such as *Sterechinus neumayeri* and *Ophionotus victoriae* both found in high abundance across a large depth range with catholic diets^66,67^, will likely thrive as niches shift and new opportunities become available. A key feature in assemblage response to disturbance shift is dispersal capability^25^; broadcast-spawning species, such as *Cnemidocarpa verrucosa*^68^, may be better able to redistribute in response to the changing environment. While species that have low reproductive rates but are sensitive to climate forcing, such as *Anoxycalx joubini* (structure-forming hexactinellid sponge) may spawn to respond to these changing conditions^69^. Generally species with low reproductive rates are likely to suffer, however this may be countered by mobile species, whose adults can adjust depth ranges through movement such as *Trematomus bernacchii* (Nototheniidae fish)^70^. Ultimately however if warming continues glaciers will retreat past grounding lines and iceberg calving rates will drop dramatically resulting in a complete reversal to low levels of iceberg disturbance across all depths^42^. This will likely form a new climax community with lower diversity and dominated by porifera (sponges) usually found in deeper water, as can be seen in small, sheltered areas of the seabed where much deeper species dominate (e.g., overhangs and caves^28^). However, in the previously high disturbance area between 10 and 30 m there may be small increases in richness and diversity, as macro-algae and their associated fauna increase.

To summarise, even though the Intermediate Disturbance Hypothesis is debated^9,14,15,71^, our results are congruent with this explanation for the Antarctic benthos disturbance-diversity pattern which can be detected because of the broad range of disturbance regimes included in this study. The consequences of the diversity-disturbance patterns within shallow Antarctic benthos will have profound impacts, particularly with glacial retreat opening new fjordic habitats and potential providing new carbon sinks and negative climate feedback loops^42^. The future of the shallow Antarctic benthos is likely to involve dramatic fluctuations in biodiversity and ecosystem functioning, and should warming continue, could ultimately lead to locally large losses in biodiversity with far-reaching implications.

## Supplementary Information

Supplementary Information.
