# Supplementary material for: Intermediate ice scour disturbance is key to maintaining a peak in biodiversity within the shallows of the Western Antarctic Peninsula
Source: Sci Rep. 2021 Aug 18;11:16712. doi: 10.1038/s41598-021-96269-9 (PMC8373922; doi:10.1038/s41598-021-96269-9)
Supplement: Supplementary file 1 — Supplementary Information. [file 41598_2021_96269_MOESM1_ESM.pdf]

Supplementary Information for

Intermediate ice scour disturbance is key to maintaining a peak in biodiversity within the Shallows of the Western Antarctic Peninsula

Authors

B. J. O. Robinson*, D. K. A. Barnes, L. J. Grange and S. A. Morley

Corresponding Author BJOR: benson@bas.ac.uk

The PDF includes:

Supplementary figures and materials 1-7

Figure S1

Three sites on a steep underwater rock slope on Adelaide Island, Western Antarctic Peninsula.

(67° 35’ S, 068° 07’W):

Site 1: LAT 67° 33.352’S, LONG 068° 11. 836’ W

Site 2: LAT 67° 34.185’S, LONG 068° 06. 060’ W

Site 3: LAT 67° 34.392’S, LONG 068° 07. 711’ W

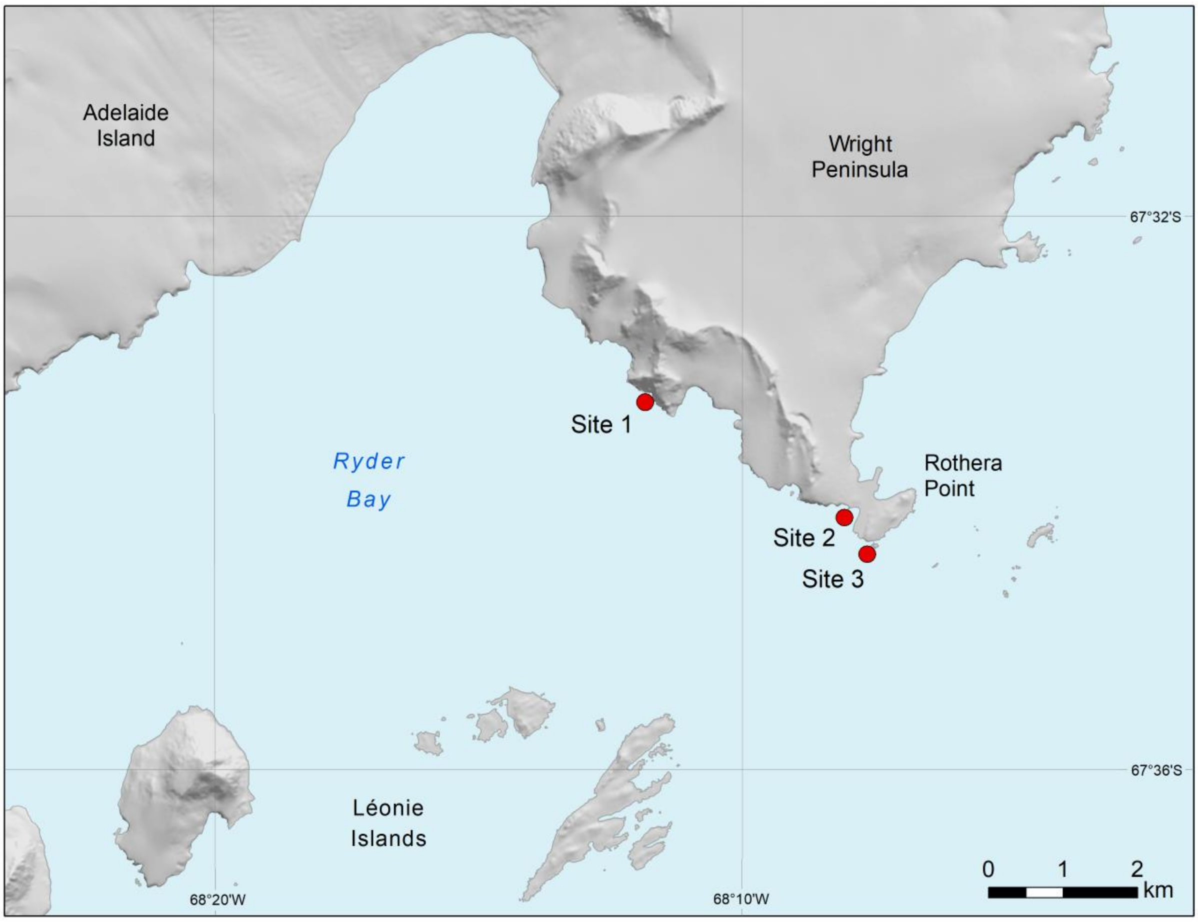

Supplementary Figure S2

Seabed depth profile recorded across all 3 sites, depths recorded using on the R.V. Nimrod echo-sounder and GPS. Each point represents an individual measurement. Multiple depth profiles were measured at each site, this figure contains a typical example of each. Plots constructed in SigmaPlot v13.0, http://www.sigmaplot.co.uk/index.php.

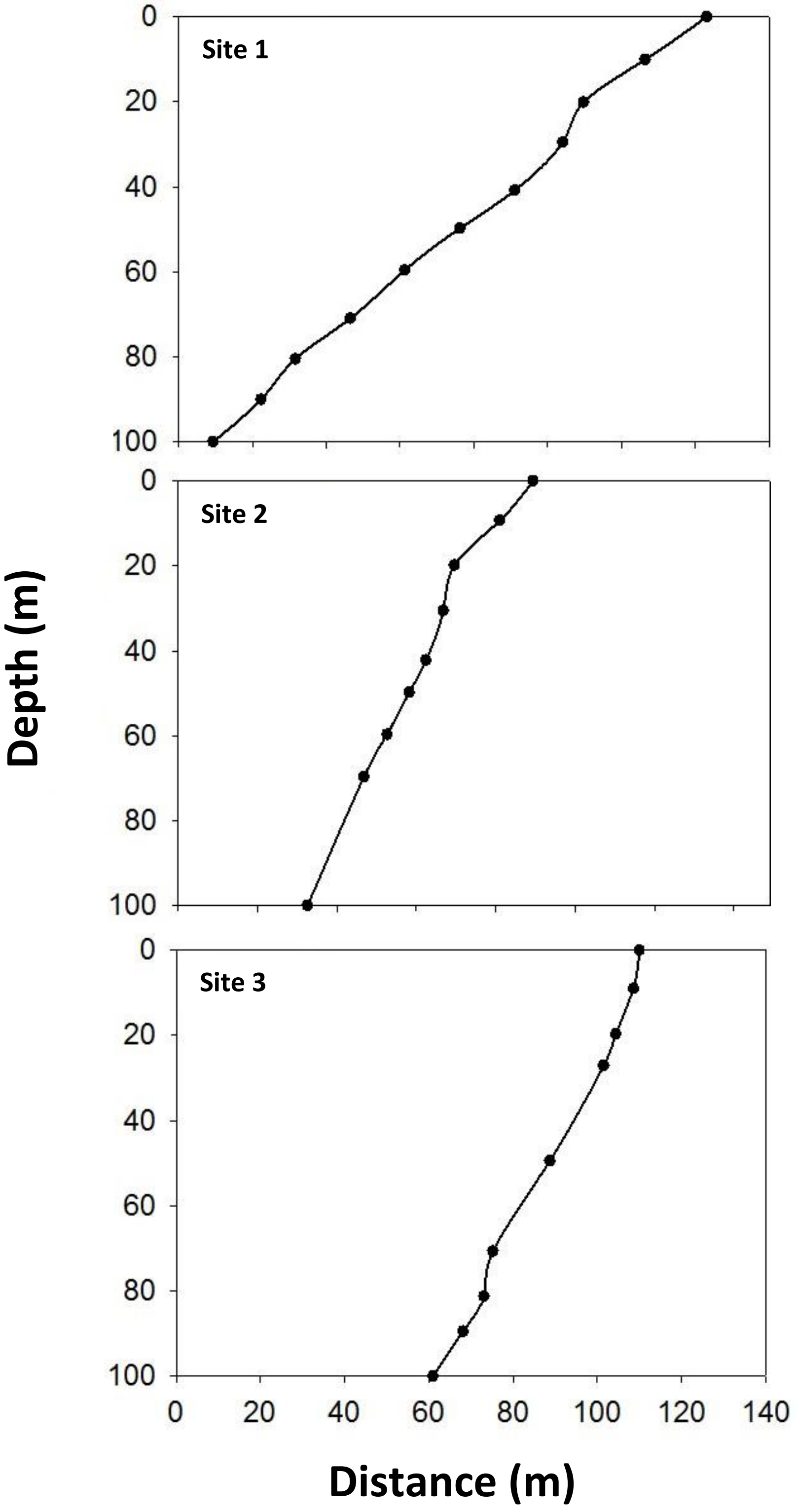

Supplementary material S3

Relationship between A) Primary productivity, B) maximum Temperature range, C) Benthic growth rate, D) Ice scour disturbance, E) average annual Salinity, F) average annual Temperature, G) average annual light levels (PAR) and depth. Environmental variables were collated from the Rothera Oceanographic and Biological Time Series (RaTS). All RaTS data was averaged across months to ensure even representation of the annual variation from 2011 to 2018. Maximum temperature range was calculated as the maximum and minimum recorded temperature from all 7 years at each specific depth. Plots constructed in SigmaPlot v13.0, http://www.sigmaplot.co.uk/index.php.

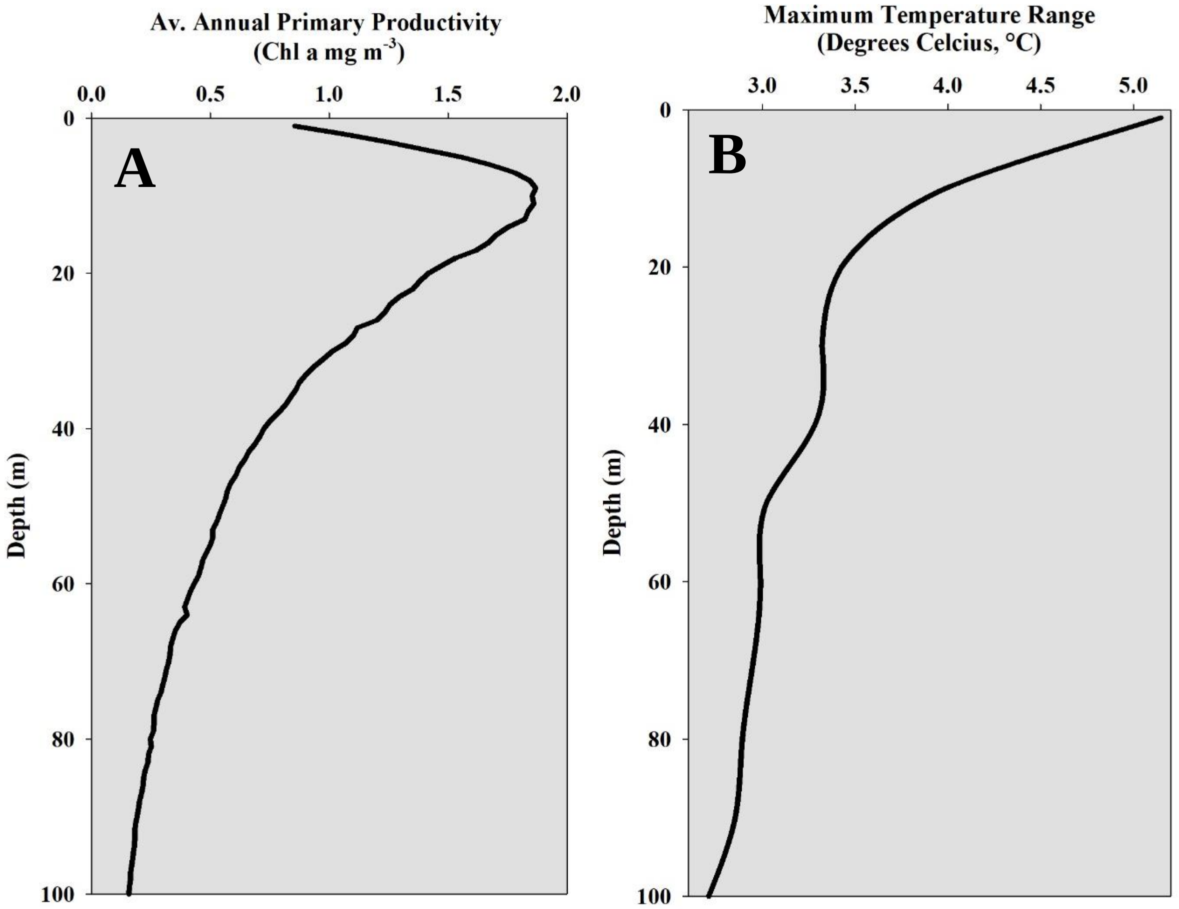

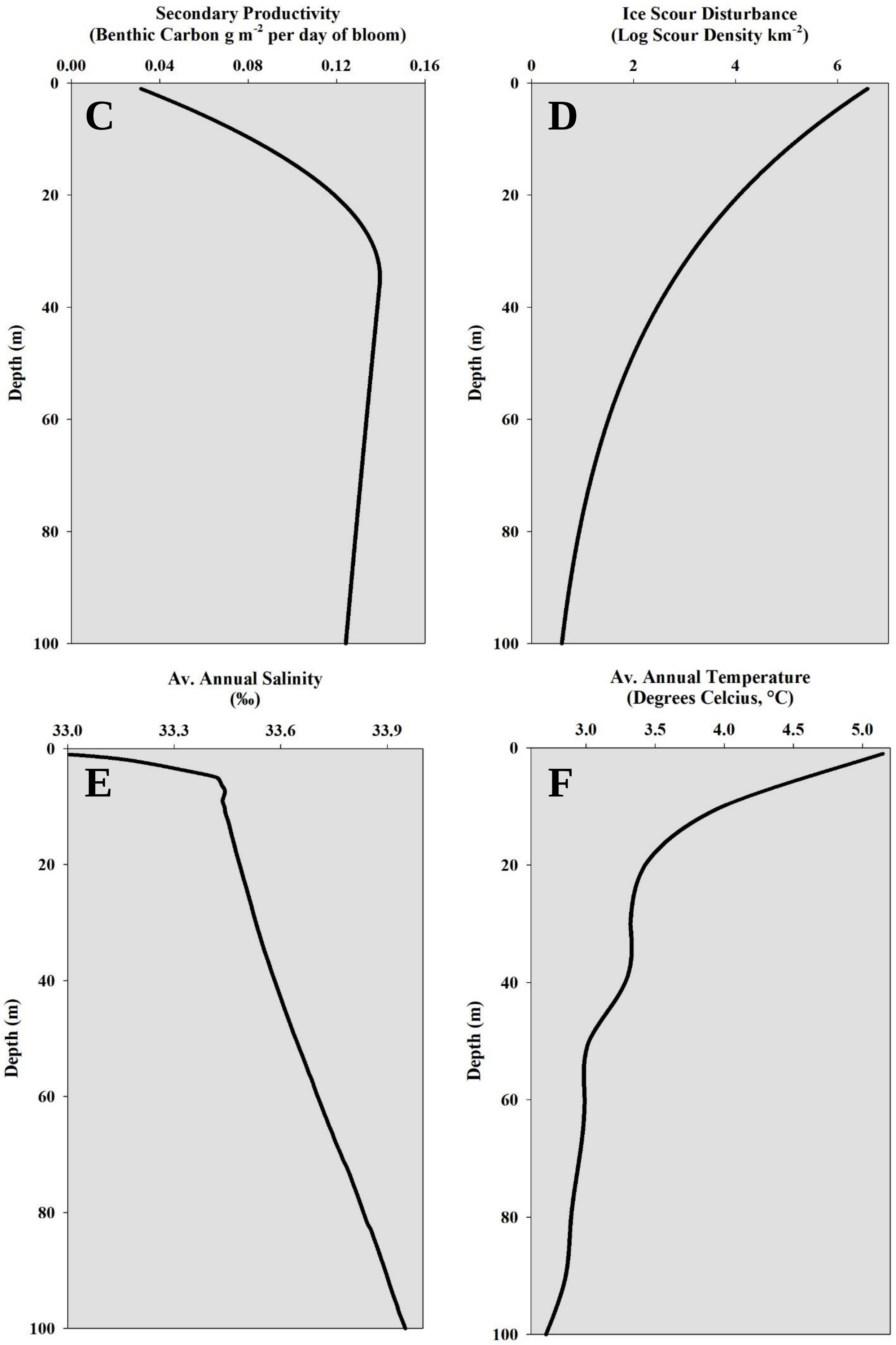
Secondary Productivity
(Benthic Carbon g m-2 per day of bloom)
0.00 0.04 0.08 0.12 0.16
0 20 40 60 80 100
Depth (m)
C
Ice Scour Disturbance
(Log Scour Density km-2)
0 2 4 6
0 20 40 60 80 100
Depth (m)
D
Av. Annual Salinity
(‰)
33.0 33.3 33.6 33.9
0 20 40 60 80 100
Depth (m)
E
Av. Annual Temperature
(Degrees Celcius, °C)
3.0 3.5 4.0 4.5 5.0
0 20 40 60 80 100
Depth (m)
F

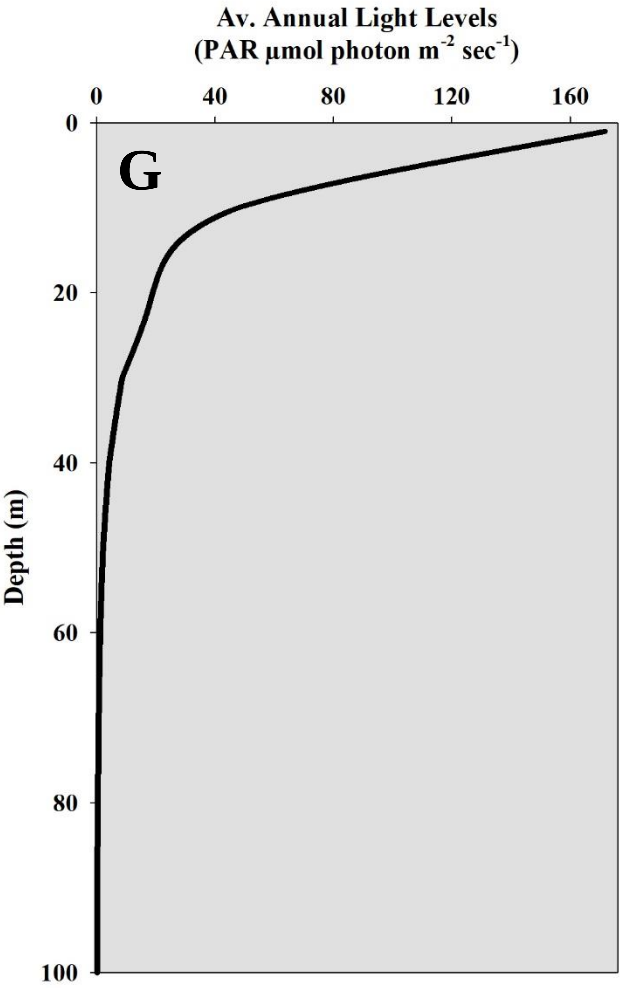

Av. Annual Light Levels
(PAR µmol photon m-2 sec-1)
0
40
80
120
160
0
20
40
60
80
100
G
Depth (m)

Supplementary material S4 – Multiple regression analysis: Site and Sediment Cover

There was significant variation detected in species richness between site 1 and the other two sites (*one-way ANOVA* $f_{(2,1497)} = 9.12, p<0.001$). This observation was driven by a marginally lower mean species richness (*-1.6 species*) at 100, and 90 m depth at site 1, which had slightly more exposed topography to ice scour. When site was included in a multiple regression model, this did not significantly improve relative model quality ($\Delta AIC=-0.588$). Sediment cover had a weak positive linear correlation with depth ($f_{(1,1499)} = 9.12, r^2=0.146, p<0.001$), but only slight correlation with ice scour disturbance ($VIF = 1.17$). Inclusion of sediment and site, measures to account for the small-scale changes in bottom composition and topography, into a multiple regression model did not significantly improve the quality of the model and were not significantly associated with species richness, in contrast to ice scour disturbance (Table 1).

Coefficient	Estimate	Std. error	t-value	p-value
Ice scour	-2.676	0.112	-23.915	<2e-16*
Site	0.683	0.411	1.661	0.0969
Sediment	0.005	0.006	0.842	0.3999

Supplementary material S5 – List of morphotypes and correspondent macro and megafauna species

**Morpho-type**	**Species Name**	**Common name**
ACT001	*Notothenia rossii*	Green fish
ACT002	*Trematomus bernacchii*	Pink Fish
ACT003	*Artedidraco micropsi*	Plunderfish
ALG001	*Plocamium cartilagineum*	Fine red Algae
ALG002	?	Pink encrusting algae
ALG003	*Palmaria decipens*	Foliose red algae
ALG004	*Lambia antarctica*	Green Algae
ALG005	*Himantotalus grandifolium*	Brown fine
ALG006	*Iridaea coradata or Gigartina skottsbergii*	Red large leaf
AMP001	*Djerboa furcipes*	Red Amphipod
ANT001	*Primnoella* sp.	Seawhip
ANT002	*Thourella* sp.	Busy Seawhip
ANT003	*Corallimorphus karinae*	Translucent anemones
ANT004	*Armadilogorgia cyathella*	Two-branch seawhip
ANT005	*Dactylanthus* sp.	Translucent squat anemone
ANT006	*Anthomastus bathyproctus*	Globular octocoral
ANT007	N/A	Small burrowing dark anemone
ANT008	*Edwardsiella ignota*	Small burrowing white anemone
ANT009	N/A	Brown small anemone
ANT010	*Edwardsiella* sp.	Small white anemone
ANT011	*Alcyonium antarcticum*	Branching yellow/orange octocoral
ANT012	*Isotealia antarctica*	Salmon anemone
ANT013	*Hormathia lacunifera*	Large white burrowing anemones
ANT014	*Stomphia selaginella*	Red/orange anemones

ANT015	*Artemidactis victrix*	Large white attached anemone
ANT016	*Gersemia antarctica*	Erect soft coral
ANT017	*Umbellula* sp.	Thin stalked anemone, large arms
ANT018	N/A	Stalked orange singular octocoral
ASC001	*Dideminidae* sp.	Pale blotchy clonial ascidian
ASC002	*Pareungyriodes arnbackae*	Long stalk, trans body
ASC003	Pyura setosa	Hairy ascidian
ASC004	*Mogula* sp.	Trans solitary ascidians
ASC005	N/A	White dot, stalked, solitary ascidian
ASC006	*Pyura discoveryi*	Brown solitary ascidian
ASC007	N/A	Yellow colonial ascidians
ASC008	N/A	Semi-orange ascidians
ASC009	*Cnemidocarpa verrucosa*	Large barrel ascidians
ASC010	N/A	Orange/red colonial
ASC011	*Ascidia* sp.	Trans yellow clump
ASC012	N/A	Vental covered ascidian
ASC013	*Dideminidae* sp.	Brown clonial ascidian
ASC014	*Sycozoa sigillinoides*	Staled white dot, elongated
ASC015	N/A	Clumps translucent ascidians
ASC016	*Ascidia challengeri*	covered ventral solitary ascidian
AST001	*Odontaster validus*	Red, pale underside
AST002	*Cryptasterias turqueti*	Pale orange soft arms, sea star
AST003	N/A	Tiny white rigid sea star

AST004	*Diplasterias brucei*	Light yellow, ridged arms, sea star
AST005	*Cycethra verrucosa*	Yellow/orange slender
AST006	*Porania antarctica*	Slightly domed sea star
AST007	*Acodontaster conspicuus*	Pale margin sea star
AST008	*Mactoptychaster accrescens*	Large thick, tapering sea star
AST009	*Odontaster meridionalis*	Slightly domed sea star
AST010	*Pteraster rugatus*	Tiny domed white
AST011	Cuenotaster involutus	Large bristle sea star
AST012	*Perknaster aurorae*	White/red large sea star
AST013	*Perknaster fuscus*	Yellow/red perknaster
AST014	*Labidiaster radiosus*	Large multi-limbed sea star
AST015	*Lophaster* sp.	Rigid white bristle sea star
AST016	*Solaster regularis*	8-Arm yellow sea star
BRA001	*Liothyrella uva*	Brachiopods
BRY001	*Carbesa ovoidea/ Nematoflustra flagellate/ Isosecuriflustra tenuis/ Isosecuriflustra angustra*	Broad foliose bryozoans
BRY002	*Cornucopina polymorpha/ Camtoplites giganteus/ Camptoplites retiformis/ Notoplites vanhoffeni/ Isosecuiflustra anagusta/ Notoplites drygalskii/ Camptoplites bicornis/ Cellaria clavata/*	Thin-branched foliose bryozoans

	Klugeflustra antarctica/ Camptoplites latus/ Notoplites tenuis/ Camptoplites tricornis	
BRY003	*Lageneschara lyrulata*	Encrusting bryozoans
BRY004	*Cellarinella watersi*	Lobed branched foliose bryozoans
BRY005	*Systenopora contrata*	Orange broad foliose bryozoans
BRY006	*Caberea darwinii*	Small curricular foliose bryozoans
BRY007	Isoschizoporella virgula	Thin lobed foliose bryozoans
BRY008	*Reteporella frigida*	Fenestrate "brandy-snap" bryozoans
BRY009	*Hornea* sp.	Skeletal branched bryozoans
BRY010	*Smittina obicullata*	Orange encrusting bryozoans
BRY011	*Fasciculipora* sp.	Calcareous branch erect bryozoans
BRY012	*Isoschizoporella similis*	Calcareous "ross-coral" bryozoans
BRY013	Carbasea curva	Red bryozoans
BRY014	*Kymella polaris*	Yellow broad byrozoan
BRY015	N/A	Large flexible fan bryozoan
BRY016	*Beania* sp.	Fuzzy encrusting bryozoans
BRY017	Neofungella sp.	Small singular globular coral

CEP001	*Adelieledone polymorpha*	Octopus
CRN001	*Promachocrinus kerguelensis*	Pale crinoid
CRN002	*Promachocrinus kerguelensis*	Dark crinoid
DEC001	*Euphausia* sp.	Semi-translucent red shrimp
DEC002	N/A	Sandy Shrimp
ECH001	*Sterechinus neumayeri*	Red sea urchin
ECH002	*Ctenocidaris gigantea*	Pencil urchin
ENT001	*Barentsia* sp.	Fuzzy on worm tube
GAS001	*Nacella concinna*	Limpet
GAS002	*Marseniopsis conica*	Orange bumpy Lamellarian gastropod
GAS003	*Iotha coppingeri*	Limpet, cap-shaped shell
GAS004	*Charcotia granulosa*	Nudibranch, translucent white rim gills
GAS005	*Marseniopsis mollis*	Yellow Lamellarian gastropod
GAS006	*Cuthona crinita*	Translucent, white tip nudibranch
GAS007	*Austrodoris kerguelensis*	Bumpy brown nudibranch
GAS008	*Newnesia* sp.	White snail
GAS009	*Marseniopsis* antarctica	Sea orange with white snout
GAS010	*Laternula elliptica*	Soft shell clam
GAS011	*Adamussium colbecki*	Scallop, purple
GAS012	*Nothoadmete tumida*	Whelk
GAS013	*Aegires albus*	All white nudibranch
GAS014	*Adamussium colbecki*	Juv. Spat Scallop, purple
GAS015	*Marseniopsis syowaensis*	Translucent sea orange
GAS016	*Limopsis* sp.	Red clam
HOL001	*Cucumaria* sp.	Orange holothurian

HOL002	*Psolus charcotii*	Pink tentacles, burrowed in sediment
HOL003	*Echinopsolus acanthocola*	Pink tentacles, epi-benthic holothurian
HOL004	*Heterocucumis steineni*	Black and white holothurian
HOL005	*Psolus dubiosus*	Orange/brown body w pink tentacles holothurian
HOL006	*Staurocucumis liouvillei*	Translucent holothurian
HOL007	*Bathyplotes bongraini*	Spikey sea pig
HYD001	*Stegella lobata*	Branched hydroid
HYD002	N/A	Yellow stylasterid hydroid
HYD003	N/A	Hydroid growing on feature worm
HYD004	*Oswaldella* sp.	Feather Hydroid
HYD005	*Cadelabrum penola*	Erect squish hydroid
ISO001	N/A	Mantis shaped, yellow isopod
ISO002	N/A	Beige isopod
NEM001	*Parbolasia corrugatus*	Parabolsia
OPH001	*Ophionotus victoriae*	Brittle star
OPH002	*Ophiosparte gigas*	Giant Brittle star
OPH003	*Ophiacantha pentactis*	Spikey white 6-arm brittle star
OPH004	*Astrotoma agassizi*	Long white armed brittle star
POL001	*Flabelligera mundata*	Large distinctive spines worm
POL002	*Perkinsiana littoralis*	Feather Worm
POL003	N/A	Calcareous straight tube
POL004	*Paralaeospira levinsi*	Circular spirorbid worm
POL005	N/A	Terebellid worms

POL006	N/A	Sandmason worms
POL007	*Chaetopterus polycheates*	Parchment worm
POL008	*Laetmonica prodicta*	Bristle Aphrodite worm
PPP001	*Nuttallochiton mirandus*	Large mantle polyplacophora
PPP002	*Callochiton steinii*	Small, rounded polyplacophore
SPO001	N/A	Yellow encrusting sponge
SPO002	*Suberites topsenti*	Globular, lobate sponge
SPO003	*Haliclona scotti*	Small, orange, tubular sponge
SPO004	N/A	Encrusting yellow sponge colonising feather worm
SPO005	N/A	Red/orange Encrusting sponge
SPO006	N/A	Pale brown globular sponge
SPO007	*Haliclona* sp.	Long yellow tubular sponge
SPO008	N/A	White discoloured sponge
SPO009	*Rossella racovitzae*	Spikey white volcano sponge
SPO010	N/A	Encrusting white sponge attached to feather worm tube
SPO011	N/A	Orange flattened sponge with fronds
SPO012	*Clathria (Axosuberites) nidificata*	Light brown bristle sponge
SPO013	*Isodictya erinacea*	Conical yellow encrusting sponge
SPO014	*Microxina benedeni*	Clavate pink sponge
SPO015	*Leucascus leptoraphis*	String sponge

SPO016	*Stylocordyla chupachups*	Lollipop sponge
SPO017	*Clionopsis* sp.	Orange textured (mucus) sponge
SPO018	*Haliclona tenella*	Translucent globular sponge
SPO019	*Cinachyra antarctica*	Round spikey golfball sponge
SPO020	N/A	Encrusting yellow/brown sponge
SPO021	*Homaxinella balfourensis*	Long stringy sponge
SPO022	N/A	2-layer translucent sponge
SPO023	*Drendrilla antarctica*	Yellow spikey erect sponge
SPO024	*Calyx arcurius*	Beige fan sponge
SPO025	*Phorbas areolatus*	Yellow/brown globular sponge
SPO026	*Anoxycalyx joubeni*	White boulder sponge
SPO027	*Sphaerotylus antarcticus*	Yellow Siphon sponge
SPO028	*Calyx shackleton*	White fan sponge
SPO029	N/A	Yellow boulder sponge
SPO030	*Mycale acerata*	Orange bobbly (slimy) sponge
SPO031	N/A	Yellow bobbly sponge
SPO032	*Polymastia invaginata*	Large siphon sponge
SPO033	N/A	White staghorn sponge
SPO034	*Isodictya setifera*	Stringy sponge
SPO035	*Guitarra antarctica*	Silt globe yellow sponge
SPO036	*Kirkpatrickia variolosa*	Red globe
SPO037	*Suberties caminatus*	Sea peach
SPO038	N/A	Fuzzy pale orange erect
PYC001	*Pycnogonum rhinoceros*	Yellow sea spider
PYC002	*Nymphon* sp.	Orange/brown sea spider

PYC003	*Colossendeis* sp.	Red sea spider
SIP001	*Golfingia margaritacea*	Spinculid/peanut worm
CTE001	*Lyrocteis flavopallidus*	Yellow sessile ctenophore

Figure S6

Macro and megafaunal composition change across depth (10-100 m), taxa transformed using square root function to reduce the influence of hyper-abundant species. The non-metric MultiDimensional Scaling (nMDS) was completed after 999 permutations using Bray-Curtis resemblance matrix. Overlay vectors superimposed in the bottom left corner display the influence of abundant taxa in the construction of the nMDS. A 2D stress value of 0.20 requires interpretation of a third dimension[1]. 3D nMDS stress values of 0.15 and resulted in a greater degree of separation particularly between 80-100 m depth. Both interpretations describe a broadly similar assemblage composition across depth. Plot constructed in Primer v7, https://www.primer-e.com/.

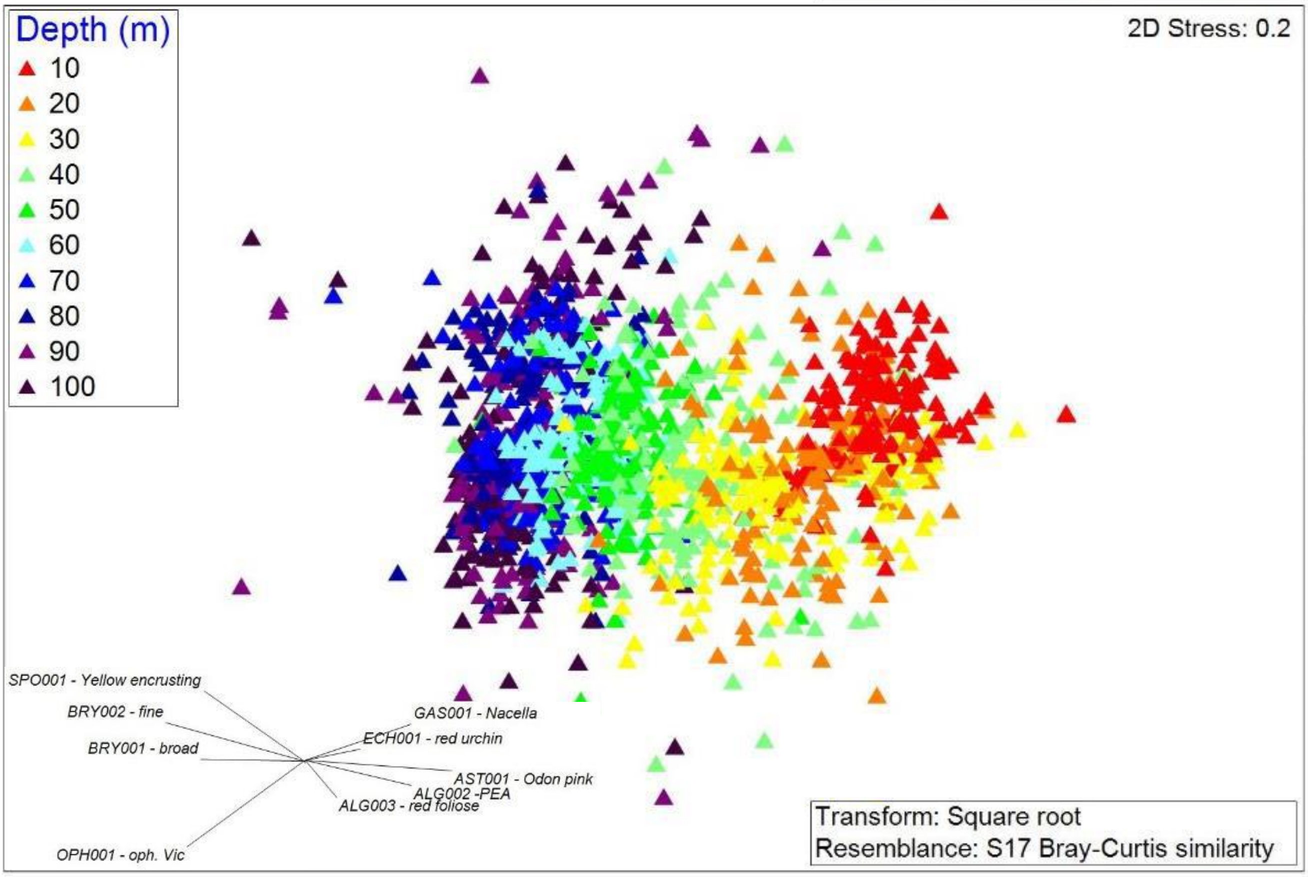

Figure S7

Relationships between biodiversity and disturbance**.** The relationships are best described non-linear regression (cubic model, black line). Gray circles are samples. Blue lines refer to 95% confidence intervals. Margelef diversity index needs to be interpreted cautiously as it does not use relative proportions in the calculations[2]. Plots constructed in SigmaPlot v13.0, http://www.sigmaplot.co.uk/index.php.

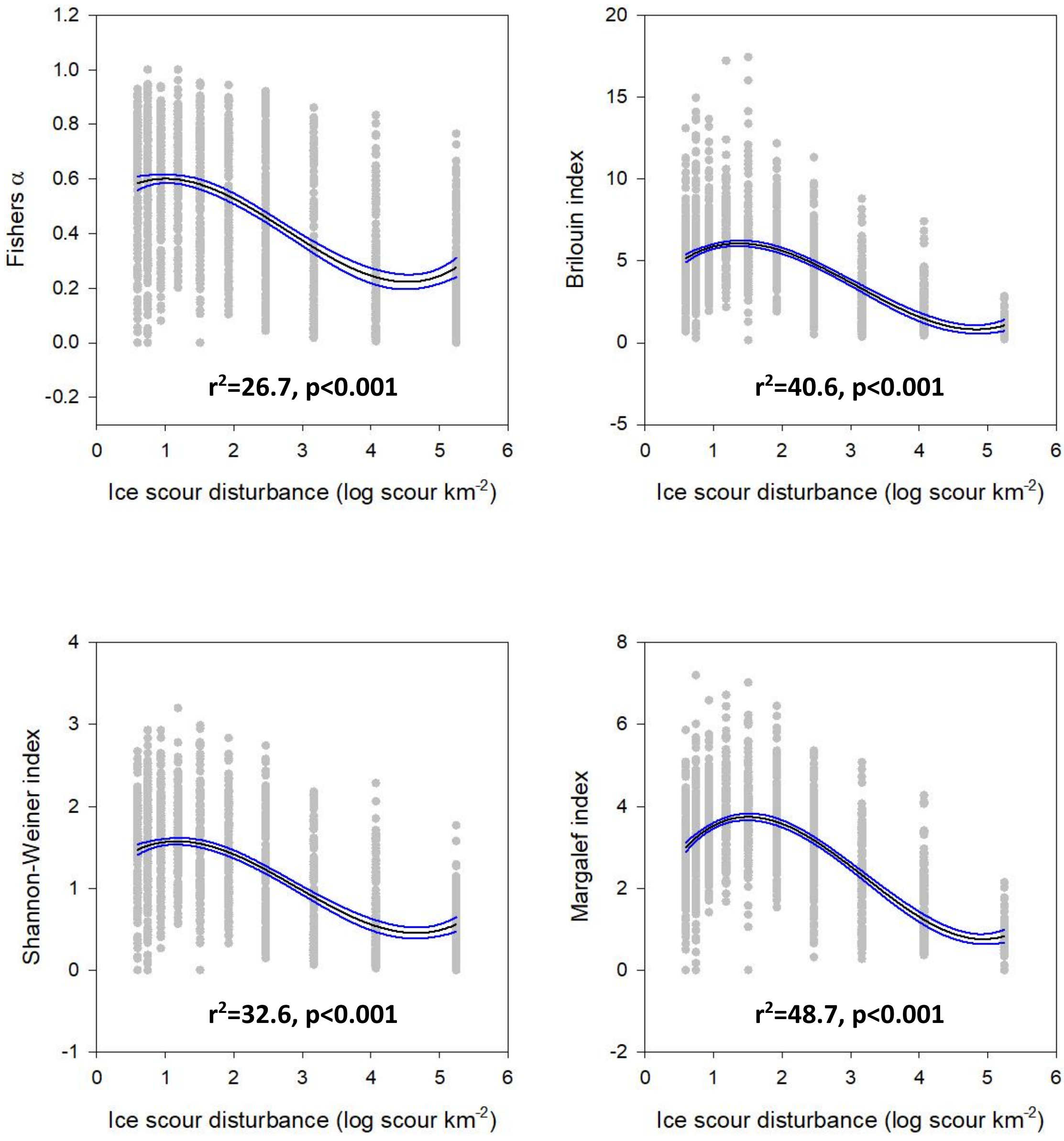

Supplementary material S8 – Growth of selected macrofauna

Growth of selected macrofauna, using growth rings of selected sessile fauna, bryozoans and spirobids, revealed a strong correlation between growth rates and standing stocks[3]. However standing stock can be strongly influenced by ice scour causing mass mortality events and removing biomass[4]. Linear and polynomial regression over-fit and residuals plot showed systematic errors. Spline regression was used instead. Plots constructed in SigmaPlot v13.0, http://www.sigmaplot.co.uk/index.php.

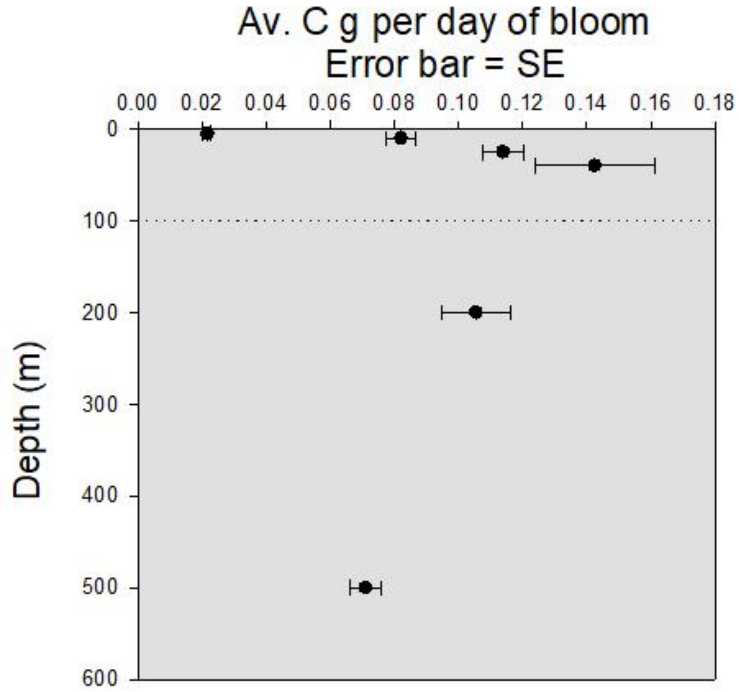

Linear Spline regression. Plot constructed in Rstudio 1.1463, https://www.rstudio.com/

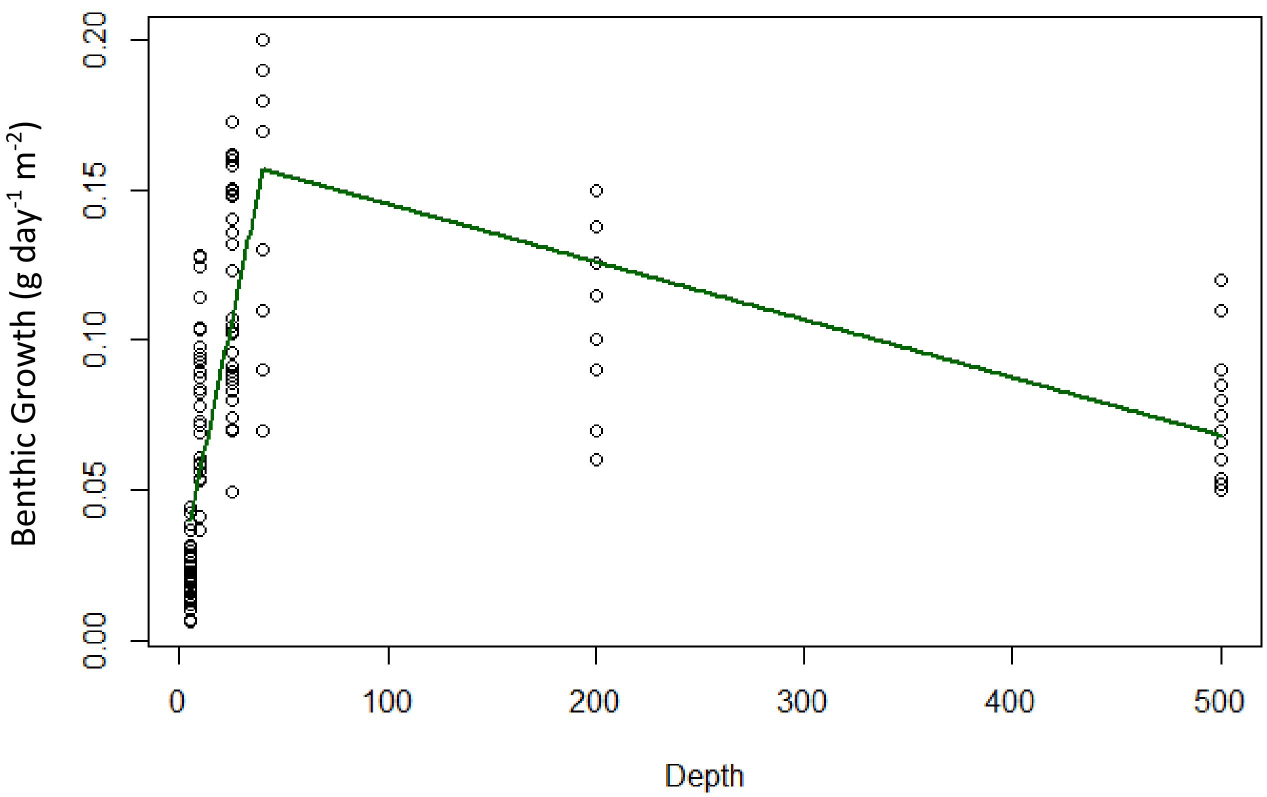


```
Call:
lm(formula = growth ~ poly(depth, 2) + depth, data = growth)

Residuals:
      Min        1Q    Median        3Q       Max
-0.082022 -0.039013 -0.006232  0.026071  0.114998

Coefficients: (1 not defined because of singularities)
                 Estimate Std. Error t value Pr(>|t|)
(Intercept)      0.074033   0.003609  20.513  < 2e-16 ***
poly(depth, 2)1  0.031547   0.042855   0.736    0.463
poly(depth, 2)2 -0.219979   0.042855  -5.133  9.5e-07 ***
depth                  NA         NA      NA       NA
---
Signif. codes:  0 ‘***’ 0.001 ‘**’ 0.01 ‘*’ 0.05 ‘.’ 0.1 ‘ ’ 1

Residual standard error: 0.04285 on 138 degrees of freedom
Multiple R-squared:  0.1631,    Adjusted R-squared:  0.151
F-statistic: 13.45 on 2 and 138 DF,  p-value: 4.625e-06
```

Residual plots for linear spline regression. plots constructed in Studio 1.1463, https://www.rstudio.com/

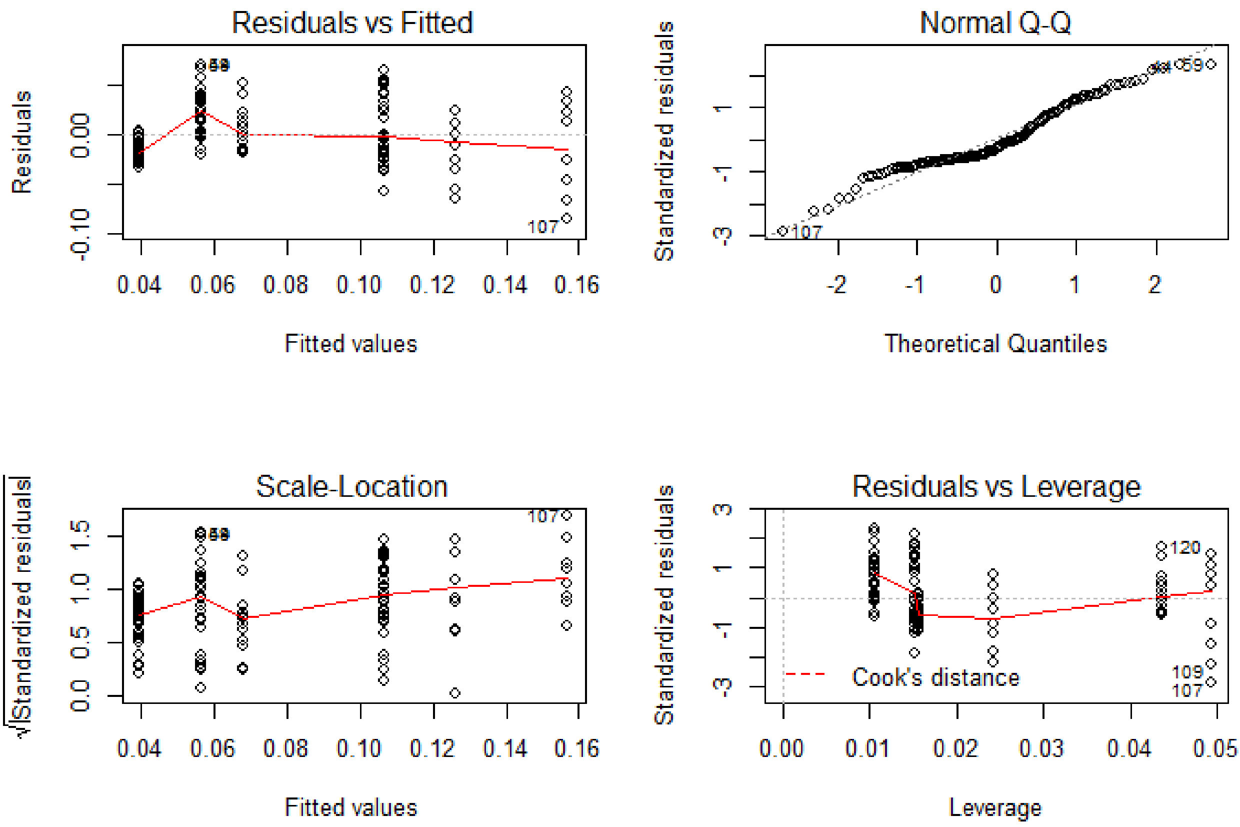

Quadratic Spline regression, plot constructed in Rstudio 1.1463, https://www.rstudio.com/

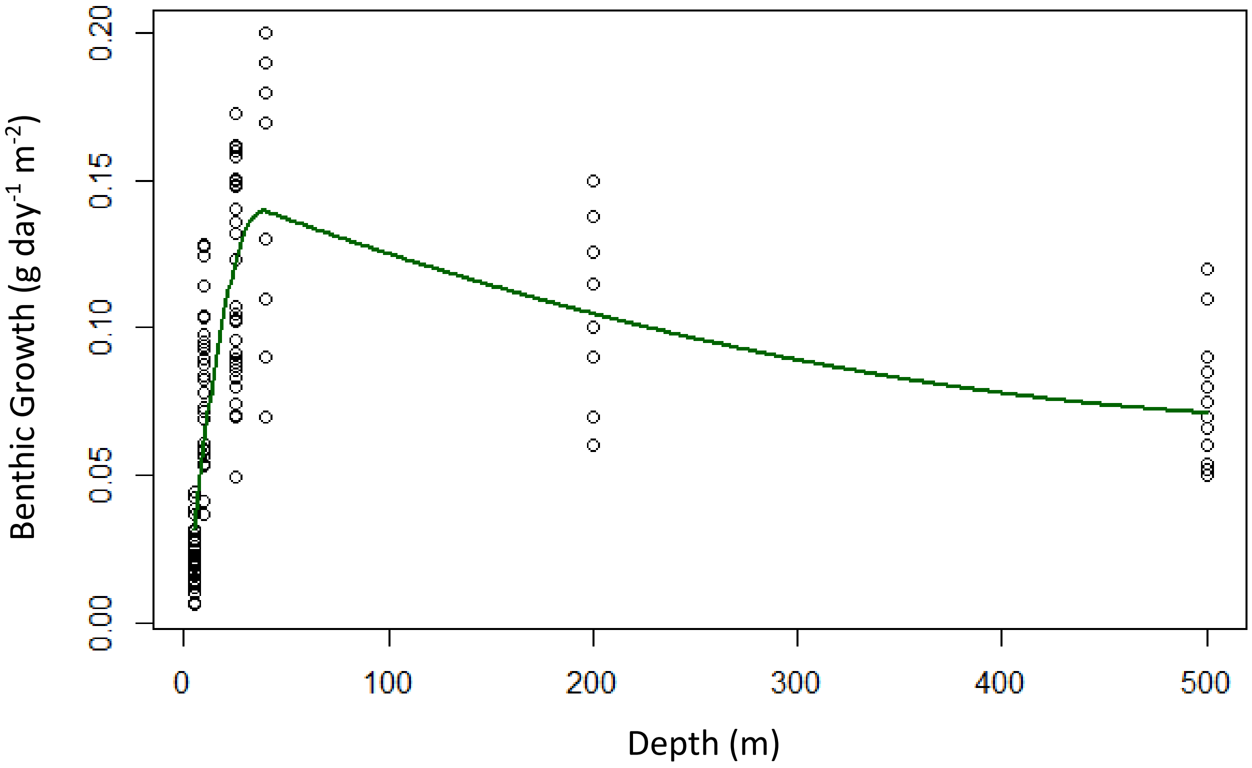


```
Call:
lm(formula = growth ~ bs(depth, degree = 2, knots = c(40)), data = growth)

Residuals:
      Min        1Q    Median        3Q       Max
-0.072315 -0.018213 -0.006901  0.021228  0.067317

Coefficients:
                                      Estimate Std. Error t value Pr(>|t|)
(Intercept)                           0.031512   0.003817   8.257 1.12e-13 ***
bs(depth, degree = 2, knots = c(40))1 0.112447   0.007717  14.572  < 2e-16 ***
bs(depth, degree = 2, knots = c(40))2 0.049681   0.020800   2.388   0.0183 *
bs(depth, degree = 2, knots = c(40))3 0.039521   0.007160   5.520 1.64e-07 ***
---
Signif. codes:  0 '***' 0.001 '**' 0.01 '*' 0.05 '.' 0.1 ' ' 1

Residual standard error: 0.02838 on 137 degrees of freedom
Multiple R-squared:  0.6355,    Adjusted R-squared:  0.6276
F-statistic: 79.63 on 3 and 137 DF,  p-value: < 2.2e-16
```

Residual plots for quadratic spline regression. Plots constructed in Studio 1.1463, https://www.rstudio.com/

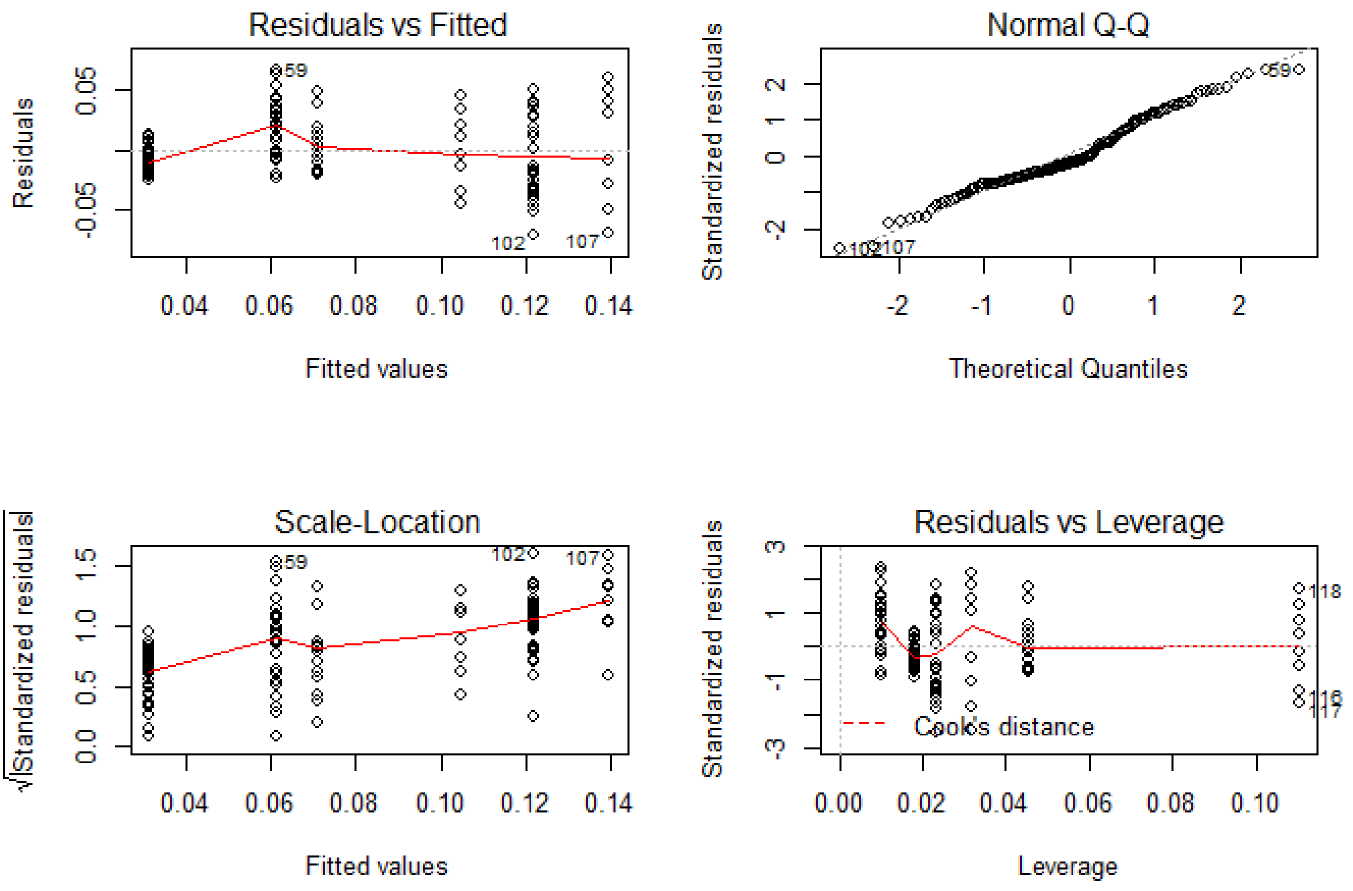

Cubic Spline Regression. Plots constructed in Studio 1.1463, https://www.rstudio.com/

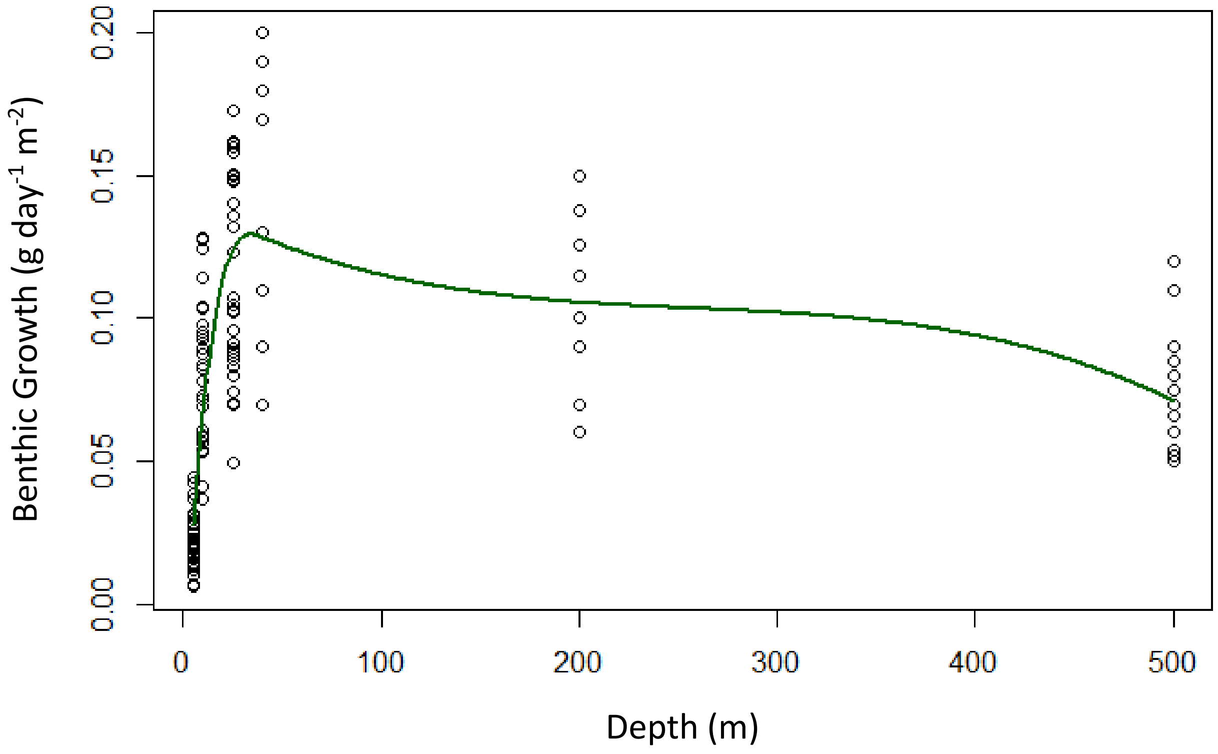


```
Call:
lm(formula = growth ~ bs(depth, degree = 3, knots = c(40)), data = growth)

Residuals:
      Min        1Q    Median        3Q       Max
-0.074375 -0.016733 -0.004998  0.016420  0.071741

Coefficients:
                                     Estimate Std. Error t value Pr(>|t|)
(Intercept)                          0.027707   0.003999   6.929 1.54e-10 ***
bs(depth, degree = 3, knots = c(40))1 0.107547   0.012623   8.520 2.67e-14 ***
bs(depth, degree = 3, knots = c(40))2 0.054786   0.130748   0.419    0.676
bs(depth, degree = 3, knots = c(40))3 0.095302   0.215408   0.442    0.659
bs(depth, degree = 3, knots = c(40))4 0.043291   0.007124   6.077 1.16e-08 ***
---
Signif. codes:  0 ‘***’ 0.001 ‘**’ 0.01 ‘*’ 0.05 ‘.’ 0.1 ‘ ’ 1

Residual standard error: 0.02765 on 136 degrees of freedom
Multiple R-squared:  0.6565,
```

Residual plots for cubic spline regression. Plots constructed in Studio 1.1463, https://www.rstudio.com/

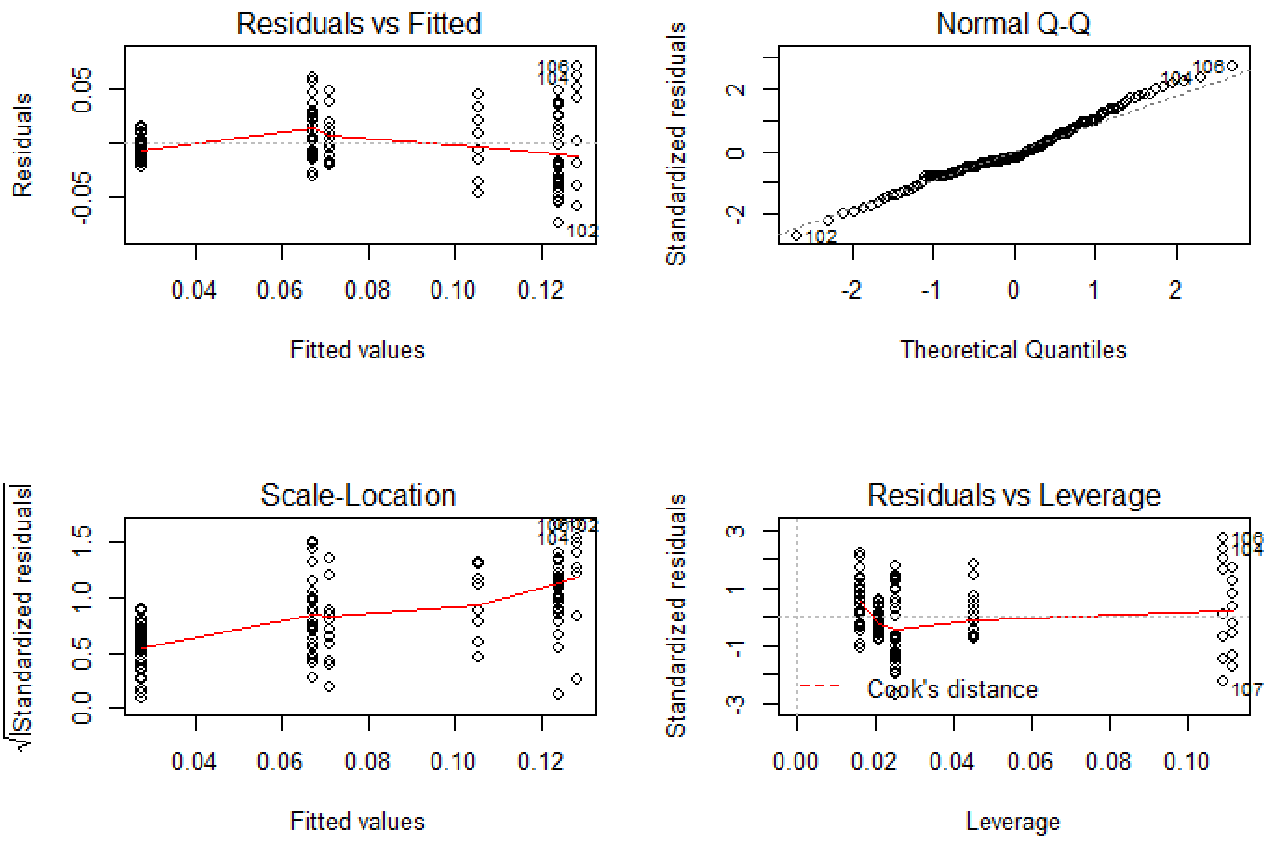

Cubic and quadratic spline regression gave very similar results, so a comparison of AIC values were used to address which approach fit the data best.

	d. f.	AIC
**Quadratic Regression**	5	-598.3894
**Cubic Regression**	6	-604.7584

As the AIC values is < 10, there is no clear distinction between the two models. As the aim is to maximise likelihood without adding redundant parameters, this study will take a parsimonious approach. Furthermore, there is greater biological evidence for a gradual and sustained decrease in benthic growth, as described by the quadratic curve, whereas decrease described between 400-500 m although minor has no biological basis. The slow and continuous decrease described by a quadratic curve, does reflect the attenuation and breakdown of marine snow into more refractory material, which would reduce growth rates. As data is sparse below 40 m depth this study will use a more parsimonious approach, relying on fewest parameters. Both show the same pattern with depth, with quadratic describing the greatest variation. Quadratic spline has a greater fit judged by AIC, is the more parsimonious approach and makes more biological sense when describing this relationship. Ultimately, however the relative difference in benthic growth between 10-100 m, does not change between quadratic or regression curves. This study used the quadratic spline regression.

Plots constructed in Studio 1.1463, https://www.rstudio.com/

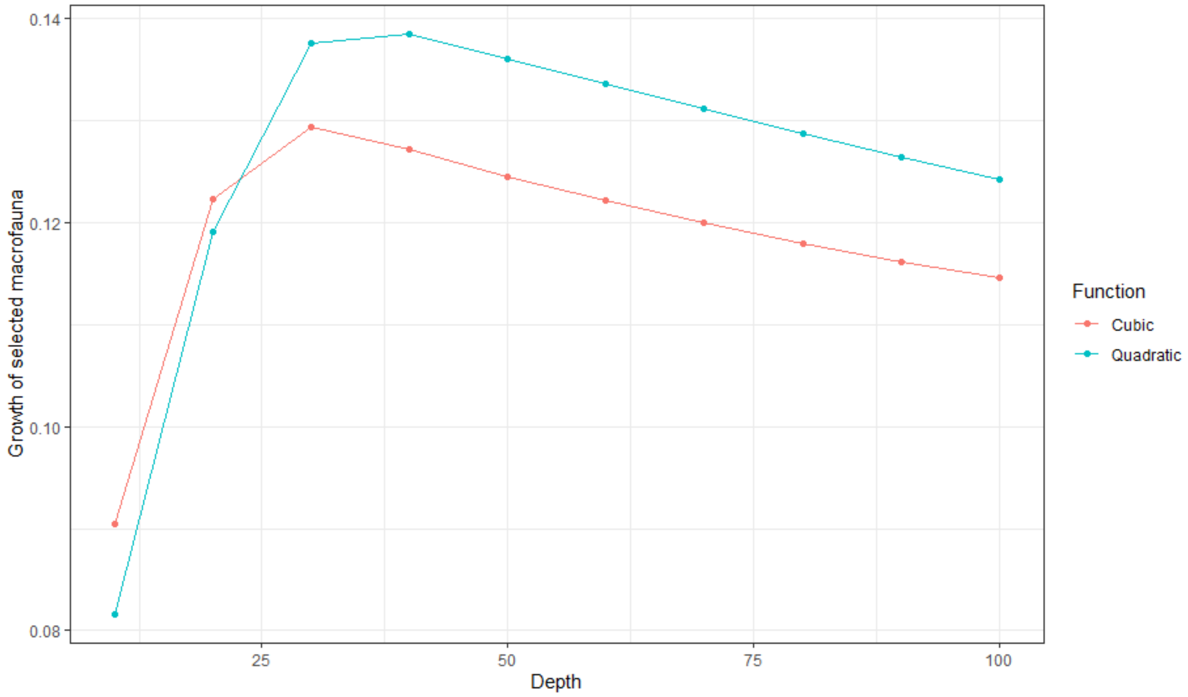

1 Clarke, K. & Gorley, R. Getting started with PRIMER v7. *PRIMER-E: Plymouth, Plymouth Marine Laboratory* **20** (2015).

2 Gamito, S. Caution is needed when applying Margalef diversity index. *Ecological Indicators* **10**, 550-551 (2010).

3 Atkinson, A., Siegel, V., Pakhomov, E. & Rothery, P. Long-term decline in krill stock and increase in salps within the Southern Ocean. *Nature* **432**, 100-103 (2004).

4 Barnes, D. K. A. & Souster, T. Reduced survival of Antarctic benthos linked to climate-induced iceberg scouring. *Nature Climate Change* **1**, 365-368, doi:10.1038/nclimate1232 (2011).
